# Association between circulating CD34-positive cell count and height loss among older men

**DOI:** 10.1038/s41598-022-11040-y

**Published:** 2022-05-03

**Authors:** Yuji Shimizu, Shin-Ya Kawashiri, Kenichi Nobusue, Fumiaki Nonaka, Mami Tamai, Yukiko Honda, Hirotomo Yamanashi, Seiko Nakamichi, Masahiko Kiyama, Naomi Hayashida, Yasuhiro Nagata, Takahiro Maeda

**Affiliations:** 1 Department of General Medicine, Nagasaki University Graduate School of Biomedical Sciences, Nagasaki, Japan; 2 Department of Cardiovascular Disease Prevention, Osaka Center for Cancer and Cardiovascular Diseases Prevention, Osaka, Japan; 3 Department of Community Medicine, Nagasaki University Graduate School of Biomedical Sciences, Nagasaki, Japan; 4 Leading Medical Research Core Unit, Nagasaki University Graduate School of Biomedical Sciences, Nagasaki, Japan; 5 Department of Islands and Community Medicine, Nagasaki University Graduate School of Biomedical Sciences, Nagasaki, Japan; 6 Department of Immunology and Rheumatology, Nagasaki University Graduate School of Biomedical Sciences, Nagasaki, Japan; 7 Nagasaki University Health Center, Nagasaki, Japan; 8 Division of Strategic Collaborative Research, Atomic Bomb Disease Institute, Nagasaki University, Nagasaki, Japan

**Keywords:** Predictive markers, Biomarkers, Cardiology, Risk factors

## Abstract

Height loss starting in middle age is reportedly significantly associated with death due to cardiovascular disease. Impaired blood flow is the main pathology in cardiovascular disease. Hematopoietic stem cells such as CD34-positive cells play an important role in maintaining the microcirculation and preventing impaired blood flow by activating endothelial repair and angiogenesis. Therefore, circulating CD34-positive cell count could be associated with height loss. To clarify the association between circulating CD34-positive cell count and height loss, we conducted a follow-up study of 363 Japanese men aged 60–69 years over 2 years. Height loss was defined as being in the highest quartile of height decrease per year. Independent of known cardiovascular risk factors, circulating CD34-positive cell count was significantly inversely associated with height loss. The fully adjusted odds ratio (OR) and 95% confidence interval (CI) of height loss for circulating CD34-positive cell count (logarithmic values) was 0.49 (0.32, 0.74). This study suggests that a lower capacity to maintain the microcirculation due to a fewer CD34-positive cells might affect height loss.

## Introduction

A previous Japanese study reported that height loss starting in middle age is significantly associated with death due to coronary heart disease or stroke but not cancer^1^. However, the mechanism underlying this association has not yet been clarified.

Recent studies have revealed an inverse association between height and cardiovascular disease^2–6^ and a positive association between height and cancer^2,7^. Those studies indicated that participants with height loss and participants with comparative short stature have a higher risk of cardiovascular disease but not cancer.

Impaired blood flow is the main pathology in cardiovascular disease. Growth of feeding vessels (angiogenesis) is the main pathological condition in cancer^8^. Hematopoietic stem cells known as CD34-positive cells contribute to angiogenesis^9,10^ and play an important role in endothelial repair^11,12^. Our previous cross-sectional studies of men aged 65–69 years showed a significant positive association between height and circulating CD34-positive cell count among those with hypertension^13^ and among those with circulating CD34-positive cell count below the median value^14^. Thus, deficiencies in the development of angiogenesis or endothelial repair (lower capacity to maintain the microcirculation) caused by lower production of CD34-positive cells lead to a higher risk of cardiovascular disease in people with short stature^15^. However, the association between circulating CD34-positive cell count and height loss has not yet been clarified.

Regarding the risk for cardiovascular disease and cancer, individuals with height loss have characteristics in common with individuals with short stature^1–7^. Therefore, height loss could be inversely associated with circulating CD34-positive cell count since circulating CD34-positive cell count indicates the capacity to maintain the microcirculation. To clarify this association, we conducted a 2-year follow-up study of 363 Japanese men aged 60–69 years who participated in a general health check-up at least twice from 2014–2017.

## Materials and methods

### Study population

The present study was performed in two locations in Nagasaki prefecture, the city of Goto and the town of Saza. Detailed descriptions were provided elsewhere^15^. The methods related to the present risk survey, including circulating CD34-positive cell measurement, also have been described elsewhere^16–18^.

Written consent forms were made available to ensure that the participants understood the objective of the study. Informed consent was obtained from all participants. All procedures performed in this study were in accordance with the ethical standards of the institutional research committee and the 1964 Declaration of Helsinki and its later amendments. Ethical approval was obtained from the Ethics Committee for Human Use of Nagasaki University. The study was approved by the Ethics Committee of Nagasaki University Graduate School of Biomedical Sciences (Project Registration Number: 14051404-13).

The study population comprised 498 male residents of Goto city and Saza town, rural communities in western Japan, aged 60–69 years who underwent an annual medical check-up from 2014–2015, which was considered the baseline evaluation. Individuals with no data on CD34-positive cells (n = 2) or serum test results (n = 2) were excluded. In order to avoid the influence of chronic inflammatory disease, subjects with a high white blood cell count (WBC count ≥ 10,000 cells/μL) (n = 5) were excluded. We also excluded 126 subjects who did not undergo an annual health check-up during the follow-up period (2016 to 2017). The remaining participants, 363 men aged 65.4 years ± 2.6 years (range, 60–69 years) were enrolled in the study. The mean follow-up period was 2.20 ± 0.53 years.

### Data collection and laboratory measurements

Medical history was ascertained by specially trained interviewers. An automatic body composition analyzer (BF-220; Tanita, Tokyo, Japan) calculated body mass index (BMI, kg/m^2^) after measuring height and weight. A current drinker was defined as an individual with ethanol intake ≥ 23 g/week. After at least 5 min of rest, blood pressure (systolic and diastolic) was measured in the sitting position using a blood pressure measuring device (HEM-907; Omron, Kyoto, Japan). A heparin sodium tube, an EDTA-2 K tube, a siliconized tube, and a sodium fluoride tube were used to collect fasting blood samples for laboratory measurements. The heparin sodium tube was used to measure CD34-positive cell count. The EDTA-2 K tube was used to measure WBC count, hemoglobin (Hb), and platelet count. The siliconized tube was used to measure high-density lipoprotein cholesterol (HDLc), triglyceride, and serum creatinine levels. The sodium fluoride tube was used to measure hemoglobin A1c (HbA1c). To measure circulating CD34-positive cell count, an automated software program on the BD FACSCanto™ II system (BD Biosciences) was used in accordance with International Society of Hematotherapy and Graft Engineering guidelines^19^.

Using standard laboratory procedures, WBC count, platelet count, and levels of Hb, HDLc, triglycerides, serum creatinine, and HbA1c were measured at SRL, Inc. (Tokyo, Japan).

Using the method proposed by a working group of the Japanese Chronic Kidney Disease Initiative^20^, the estimated glomerular filtration rate (eGFR) was calculated: eGFR (mL/min/1.73 m^2^) = 194 × (serum creatinine (enzyme method))^-1.094^ × (age)^-0.287^.

### Statistical analysis

To clarify the influence of age on circulating CD34-positive cell count and height loss, simple correlation analysis was performed.

Clinical characteristics of the study participants were compared by circulating CD34-positive cell count tertile: T1 (Low), < 0.78 cells/μL; T2, 0.78–1.31 cells/μL, and T3 (High), ≥ 1.32 cells/μL. Data are presented as means ± standard deviation (SD) or n (%), except for triglycerides. Since triglyceride values had a skewed distribution, they were expressed based on the median (interquartile range). Trend tests were also performed.

Height loss was defined as being in the highest quartile of height decrease per year. Logistic regression models were used to calculate odds ratios (ORs) and 95% confidence intervals (95% CIs) to determine the association between circulating CD34-positive cell count and height loss.

Adjustment for confounding factors was made in three ways. In Model 1, we adjusted only for age. In Model 2, we adjusted for age plus known cardiovascular risk factors, namely: BMI, systolic blood pressure (SBP), alcohol consumption status [never, former, or current [weekly alcohol consumption (23–45 g/week, 46–68 g/week, > 68 g/week)], smoking status (never, former, current), HDLc, triglycerides, HbA1c, and eGFR. Model 3 adjusted only for age, WBC count, Hb, platelet count, and height at baseline because they could act as confounding factors in the present study. CD34-positive cells are immature hematological precursors. WBC count could be associated with bone density loss^21^ while Hb could prevent height loss^22^. In conjunction with circulating CD34-positive cells, platelets contribute to endothelial repair^12^ and high platelet count is associated with low bone density^23^. In addition, height at baseline could influence the production of WBCs^24^, Hb^25,26^, platelets^27^, and CD34-positive cells^13,14^.

For sensitivity analysis, we re-ran analyses of the association between circulating CD34-positive cell count and height loss when height loss was defined as being in the highest quintile of height decrease per year.

We also performed simple correlation analysis and multiple linear regression analysis to clarify the correlation between circulating CD34-positive cell count and height loss as a continuous variable (mm/year). In multiple linear regression analysis, two models were constructed. The first model (Multivariable model 1) adjusted for age, BMI, SBP, current drinker status, current smoker status, HDLc, triglycerides, HbA1c, and eGFR. Multivariable model 2 adjusted for age, hematological parameters, and height at baseline. Logarithmic transformation was performed because circulating CD34-positive cell count and triglycerides had skewed distributions.

All statistical analyses were performed with SAS for Windows (version 9.4; SAS Inc., Cary, NC). Values of p < 0.05 were regarded as statistically significant.

## Results

### Characteristics of the study population

Among the study population, age was not significantly correlated with circulating CD34-positive cell count or height loss. The simple correlation coefficient for age and the logarithmic value of circulating CD34-positive cell count was 0.003 (p = 0.951). The correlation coefficient for age and height loss as a continuous variable was 0.02 (p = 0.656).

The characteristics of the study population by circulating CD34-positive cell count are shown in Table 1. Circulating CD34-positive cell count was significantly positively associated with known hematological parameters such as platelet count, Hb, and WBC count. Circulating CD34-positive cell count was also significantly positively associated with BMI and triglycerides and inversely associated with HDLc.

**Table 1:** Characteristics of study population by circulating CD34-positive cell levels. Values were mean ± standard deviation or n(%). *a: Values were median [first quartile, third quartile]. *b: Logarithmic transformation were performed to evaluate *p*. SBP: systolic blood pressure. DBP: diastolic blood pressure. eGFR: estimated glomerular filtration rate. CD34-positive cell count tertiles were T1 (Low), < 0.78 cells/μL; T2, 0.78–1.31 cells/μL, and T3 (High), ≥ 1.32 cells/μL.

	CD34-positive cell levels (tertiles)			*p*
	T1 (Low)	T2	T3 (High)	
No. of participants	121	122	120	
Age	65.2 ± 2.5	65.6 ± 2.7	65.4 ± 2.8	0.477
Platelets, × 10^4^/μL	20.6 ± 5.3	22.1 ± 4.5	23.5 ± 5.0	< 0.001
Hemoglobin, g/dL	14.3 ± 1.3	14.4 ± 1.0	14.9 ± 1.1	< 0.001
White blood cell, cells/μL	4718 ± 1121	5472 ± 1234	6309 ± 1272	< 0.001
Height at baseline, cm	165.2 ± 5.7	164.4 ± 5.3	164.9 ± 5.3	0.532
Body mass index (BMI), kg/m^2^	22.8 ± 3.1	23.0 ± 3.0	24.0 ± 2.5	0.002
SBP, mmHg	130 ± 18	131 ± 18	134 ± 18	0.267
DBP, mmHg	77 ± 13	77 ± 12	79 ± 10	0.144
Current drinker, %	65.3	70.0	70.0	0.681
Current smoker, %	20.7	23.0	25.8	0.637
HDL-cholesterol (HDLc), mg/dL	59 ± 13	56 ± 16	53 ± 12	0.004
Triglycerides, mg/dL	87 [63, 125]*^a^	101 [70, 130]*^a^	111 [83, 136]*^a^	0.003*^b^
Glycated hemoglobin (HbA1c), %	5.6 ± 0.5	5.7 ± 0.6	5.8 ± 0.7	0.092
eGFR, mL/min/1.73m^2^	71.3 ± 15.3	72.5 ± 15.6	73.5 ± 12.9	0.501

### Association between height loss and circulating CD34-positive cell count

Table 2 shows ORs and 95% CIs for the association between height loss and circulating CD34-positive cell count. Circulating CD34-positive cell count was significantly inversely associated with height loss; the age-adjusted OR and 95% CI of height loss for circulating CD34-positive cell count (logarithmic values) was 0.48 (0.32, 0.71). This association remained even after further adjustment for known cardiovascular risk factors (0.49, (0.32, 0.74)). In addition, hematological parameters were significantly positively associated with circulating CD34-positive cell count. The association remained significant in Model 3 that adjusted for known hematological parameters (0.45 (0.28, 0.72)).

**Table 2:** Association between height loss and circulating CD34-positive cell.

	CD34-positive cell levels (tertiles)			*p*	CD34-positive cell (Logarithmic values)
	T1 (Low)	T2	T3 (High)		
No. of participants	121	122	120		
No. of height loss case (%)	40 (33.1)	36 (29.5)	14 (11.7)		
Model 1	Ref	0.85 (0.49, 1.46)	0.27 (0.14, 0.53)	< 0.001	0.48 (0.32, 0.71)
Model 2	Ref	0.89 (0.51, 1.54)	0.28 (0.14, 0.55)	< 0.001	0.49 (0.32, 0.74)
Model 3	Ref	0.82 (0.46, 1.45)	0.24 (0.11, 0.52)	< 0.001	0.45 (0.28, 0.72)

Ref: reference. Model 1, adjusted only for age. Model 2, adjusted further for age plus body mass index (BMI), systolic blood pressure (SBP), alcohol consumption (never-, former-, current- [23–45 g/week, 46–68 g/week, 68 g/week <], smoking status (never-, former-, current), HDL-cholesterol (HDLc), triglycerides, glycated hemoglobin (HbA1c), and estimate glomerular filtration rate (eGFR). Model 3, adjusted only for age, hematological parameters (white blood cell count, platelet count, and hemoglobin), and height at baseline. CD34-positive cell count tertiles were T1 (Low), < 0.78 cells/μL; T2, 0.78–1.31 cells/μL, and T3 (High), ≥ 1.32 cells/μL.

### Correlation between height loss as a continuous variable and circulating CD34-positive cell count adjusted for cardiovascular risk factors

Table 3 shows the correlation between height loss as a continuous variable and known cardiovascular risk factors associated with circulating CD34-positive cells. Independent of known confounding variables, circulating CD34-positive count was significantly inversely associated with height loss as a continuous variable. No significant correlations between height loss and other variables were found.

**Table 3:** Correlation between height loss as a continuous variable and circulating CD34-positive cell count adjusted for cardiovascular risk factors.

	Height loss (mm/year)			
	Simple	Multivariable 1		
	r (p)	Β	β	*p*
Age	0.02 (0.656)	0.001	0.01	0.860
Body mass index (BMI)	−0.04 (0.399)	−0.002	−0.02	0.695
Systolic blood pressure (SBP)	−0.03 (0.590)	−0.0001	−0.01	0.918
Current drinker	−0.03 (0.554)	−0.01	−0.01	0.831
Current smoker	−0.03 (0.543)	−0.02	−0.03	0.624
HDL-cholesterol	−0.04 (0.486)	−0.001	−0.06	0.332
Triglycerides*^a^	0.01 (0.864)	0.02	0.04	0.544
Glycated hemoglobin (HbA1c)	−0.002 (0.964)	0.005	0.01	0.844
estimated glomerular filtration rate (eGFR)	−0.06 (0.225)	−0.001	−0.04	0.440
CD34-positive cell*^a^	−0.20 (< 0.001)	−0.09	−0.22	< .0001

r: simple correlation coefficient. Β: parameter estimate. β: standardized parameter estimate. Multivariable 1, adjusted further for age plus body mass index (BMI), systolic blood pressure (SBP), current-drinker, current-smoker, HDL-cholesterol, triglycerides, glycated hemoglobin (HbA1c), and estimated glomerular filtration rate (eGFR). *a: Logarithmic transformation were performed.

Table 4 shows the correlation between height loss as a continuous variable and hematological parameters including circulating CD34-positive cell count. Simple correlation analysis showed that Hb and circulating CD34-positive cell count were significantly inversely associated with height loss. After adjusted for those variables and height at baseline, Hb was no longer significant but circulating CD34-positive cell count remained significant.

**Table 4:** Correlation between height loss as a continuous variable and circulating CD34-positive cell count adjusted for hematological parameters and height at baseline.

	Height loss (mm/year)			
	Simple	Multivariable 2		
	r (p)	Β	β	*p*
Age	0.02 (0.656)	0.001	0.01	0.814
Platelets	−0.09 (0.084)	−0.003	−0.06	0.260
Hemoglobin	−0.11 (0.045)	−0.02	−0.07	0.168
White blood cell	−0.08 (0.151)	0.00001	0.06	0.373
Height at baseline	0.004 (0.943)	−0.0002	−0.004	0.940
CD34-positive cell*^a^	−0.20 (< 0.001)	−0.08	−0.20	0.001

r: simple correlation coefficient. Β: parameter estimate. β: standardized parameter estimate. Multivariable 2, adjusted only for age, hematological parameters (white blood cell, platelets, hemoglobin), and height at baseline. *a: Logarithmic transformation were performed.

### Sensitivity analysis

To assess sensitivity, we performed the main analyses with height loss defined as being in the highest quintile of height decrease per year, as in our previous study^22^. We obtained essentially the same results. The ORs and 95% CIs for height loss with circulating CD34-positive cell count (logarithmic values) were 0.52 (0.34, 0.79) for Model 1, 0.51 (0.32, 0.79) for Model 2, and 0.54 (0.32, 0.89) for Model 3.

## Discussion

The major finding of this study with elderly men is that circulating CD34-positive cell count is inversely associated with height loss. A previous study with Japanese workers aged 40–74 years revealed a significant inverse association between Hb and height loss defined as either being in the highest quartile or quintile of height decrease per year among men with BMI < 25 kg/m^2^ but not among men with BMI ≥ 25 kg/m^2^^22^. In the present study, simple correlation analysis showed that Hb is slightly but significantly inversely correlated with height loss. However, the correlation was not significant after adjusting for age, other known hematological parameters, and height at baseline.

In the present study, we found further evidence that, independent of BMI and Hb, circulating CD34-positive cell count is inversely associated with height loss. However, the mechanisms explaining this association have not yet been clarified.

The major causes of height loss in elderly men are intervertebral disc degeneration and vertebral fractures associated with osteoporosis. Although the development of angiogenesis has been observed in degenerative intervertebral disc lesions^28^, angiogenesis is not a major course of intervertebral disc degeneration.

The regulation of angiogenesis by hypoxia is an important component of homeostatic mechanisms that link vascular oxygen supply to oxygen demands^29^. Since hypoxia accelerates intervertebral disc degeneration^30,31^, inadequate angiogenesis related to lower adaptability to hypoxia might play an important role in the development of intervertebral disc degeneration. In addition, bone is a highly vascularized tissue, and dysregulation of the microcirculation is associated with many bone diseases, including osteoporosis^32^. Therefore, less angiogenesis related to low levels of circulating CD34-positive cells could be associated with height loss due to a higher risk of intervertebral disc degeneration and osteoporosis.

In older men, height loss is independently associated with an increased risk of all-cause mortality and coronary heart disease^33^ while a higher number of circulating CD34-positive cells is inversely associated with all-cause and cardiovascular mortality^34,35^. Therefore, the present results showing a significant inverse association between circulating CD34-positive cell count and height loss can help clarify the potential mechanism for the association between height loss and cardiovascular disease.

Measurement of CD34-positive cells requires fresh samples, analyzed within 24 h after blood collection. Approximately 30 min are required to process one sample. Since a maximum of 20 samples could be processed for CD34-positive cell measurement each day, measuring CD34-positive cell count is hard to realize in a general population study. Therefore, we limited the measurement of CD34-positive cell count to men aged 60–69 years who participated in a general health check-up in the city of Goto and the town of Saza. Detailed descriptions have been described elsewhere^15^.

The potential limitations of this study warrant consideration. Intervertebral disc degeneration and vertebral fractures associated with osteoporosis might play an important role in height loss among adults, but those data were not available to us, as in our previous study^22^. Many intervertebral disc degeneration cases are pain free^36^. Most of the vertebral fracture cases are asymptomatic and diagnosed incidentally^37^. To identify those diseases among general population, plain radiographs, computed tomography (CT), or magnetic resonance imaging (MRI) are necessary. An efficient cutoff point to define height loss has not been established. In the present study, we defined height loss as being in the highest quartile of height decrease per year. However, our sensitivity analysis based on quintile of height decrease per year showed essentially the same associations. Furthermore, a significant inverse correlation was also observed between height loss as a continuous variable and circulating CD34-positive cell count. Oxidative stress and hypoxia might play an important role in the present associations. However, we had no data to evaluate oxidative stress and hypoxia. Further epidemiological investigations with data on hypoxia inducing factor, superoxide dismutase, and 8-hydroxydeoxyguanosine are required. Due to financial and technical reasons, we limited the measurement of CD34-positive cell count to men aged 60–69 years. Further investigation with larger sample could be informative. Sex steroid hormone could influence on survival of CD34-positive cell^38^ and bone size^39^. However, as like previous studies that reported the association between height loss and cardiovascular disease^1,33^, we have no data of sex steroid hormone. Furter investigation with data of sex steroid hormone is necessary.

In conclusion, circulating CD34-positive cell count is inversely associated with height loss among elderly men. This study indicates that a lower capacity to maintain the microcirculation due to a fewer of CD34-positive cells might affect height loss.
